# The Potential Role of Mitochondrial Acetaldehyde Dehydrogenase 2 in Urological Cancers From the Perspective of Ferroptosis and Cellular Senescence

**DOI:** 10.3389/fcell.2022.850145

**Published:** 2022-04-20

**Authors:** Weizhen Zhu, Dechao Feng, Xu Shi, Qiang Wei, Lu Yang

**Affiliations:** Department of Urology, Institute of Urology, West China Hospital, Sichuan University, Chengdu, China

**Keywords:** mitochondrial acetaldehyde dehydrogenase 2, urological cancers, ferroptosis, epigenetic alterations, proteostasis, mitochondrial dysfunction, cellular senescence

## Abstract

Overproduction of reactive oxygen species (ROS) and superlative lipid peroxidation promote tumorigenesis, and mitochondrial aldehyde dehydrogenase 2 (ALDH2) is associated with the detoxification of ROS-mediated lipid peroxidation-generated reactive aldehydes such as 4-hydroxy-2-nonenal (4-HNE), malondialdehyde, and acrolein due to tobacco smoking. ALDH2 has been demonstrated to be highly associated with the prognosis and chemoradiotherapy sensitivity of many types of cancer, including leukemia, lung cancer, head and neck cancer, esophageal cancer, hepatocellular cancer, pancreatic cancer, and ovarian cancer. In this study, we explored the possible relationship between ALDH2 and urological cancers from the aspects of ferroptosis, epigenetic alterations, proteostasis, mitochondrial dysfunction, and cellular senescence.

## ALDH2: Structure, Function and Molecular Genetics

Acetaldehyde dehydrogenase-2 (ALDH2), encoded by a nuclear gene located on chromosome 12q24 (Raghunathan et al., 1988), is an enzyme rich in mitochondria with the activity of dehydrogenase, reductase and esterase (Mukerjee and Pietruszko, 1992; Chen and Stamler, 2006). ALDH2 is composed of 4 ∼56 kDa identical subunits, each of which mainly consists of the oligomerization domain, the catalytic domain, and the NAD+ -binding domain. ALDH2 provides protective effects not only by mediating ethanol metabolism and catalyzing the decomposition of acetaldehyde into nontoxic acetic acid but also by metabolizing other toxic short-chain fatty aldehydes and aromatic aldehydes (Klyosov, 1996). Furthermore, biochemical processes including decomposing endogenous aldehydic products, such as 4-HNE, malondialdehyde (MDA) and acrolein, derived from lipid peroxidation and tobacco smoking are associated with ALDH2 (Chen et al., 2010). The gene encoding ALDH2 is highly polymorphic. The most frequent point mutation, called ALDH2*2, occurs when an adenine is replaced by a guanine (rs671) at nucleotide 1459. Mutation of the ALDH2*2 enzyme disturbs the stabilization of hydrogen bonds and leads to damage to the structure of the NAD+ -binding site and several other catalytically important residues, ultimately resulting in enzyme inactivation (Li et al., 2016). Moreover, the combination of the mutant type (ALDH2*2) and the wild type, called AlDH2 *1/*2, can also result in a decrease in the catalytic activity of ALDH2 (Wang et al., 2002). It was reported that the activity of AlDH2 *1/*2 varied with the amount of AlDH2 *1/*2 in the ALDH2 tetramer, possibly reducing ALDH2 activity by 60%–80% compared to the wild-type (Weiner et al., 2001).

### ALDH2 and Cancer

Increasing body of evidence has revealed a link between the ADLH2*2 genotype and many age-related, life-shortening diseases, such as cancers and osteoporosis (Chen et al., 2014). In upper aerodigestive track (UADT) cancers, Yokoyama et al. first reported a significant high risk of esophageal cancer in ALDH2*2 carriers (Yokoyama et al., 1996). Multiple original studies, reviews and meta-analyses and from Asia also found that the mutation of ALDH2*2 and the deficiency of ALDH2 enzyme activity were commonly implicated in UADT cancers due to the increased DNA damage induced by acetaldehyde cancers (Matsuda et al., 2006; Asakage et al., 2007; Yang et al., 2007; Hiyama et al., 2008; Ding et al., 2009; Lee et al., 2009; Cadoni et al., 2012). The ALDH2 polymorphism was also reported to be linked with an increased risk of gastrointestinal cancer including gastric cancer, pancreatic cancer, hepatocellular cancer, and colorectal cancer (Miyasaka et al., 2005; Li et al., 2016). Unexceptionally, among urological cancers, it was found that heterozygous ALDH2 carriers had a higher risk of bladder cancer (BCa) (Masaoka et al., 2016). And low expression of ALDH2 was related to lower overall survival in upper tract urothelial carcinoma (Wu et al., 2014). Furthermore, as an age-related disease, prostate cancer (PCa) is one of the most common malignant tumors in European and American senior citizens and is also the main cause of death (Siegel et al., 2021). In particular, it was found that the rs671 allele of the ALDH2 gene was associated with human longevity (Park et al., 2009), indicating a potential link between PCa and ALDH2. We summarized the reaches investigating the effects of ALDH2 in urological cancers in this study in Table 1.

**TABLE 1:** The effects of ALDH2 on urological cancers in this study.

Mechanisms	Tumor type	References
Ferroptosis	BCa	Masaldan et al. (2018)
	RCa	Torti and Torti (2013); Masaldan et al. (2018)
	PCa	Yu et al. (2017); Masaldan et al. (2018); Wu et al. (2019)
Epigenetic alterations	BCa	Jerónimo et al. (2004); Sato et al. (2012); Juengel et al. (2013); Pinkerneil et al. (2016); Juengel et al. (2017); Jylhävä et al. (2017); Pinkerneil et al. (2017)
	RCa	Maegawa et al. (2010); Mao et al. (2011); Horvath (2013); Ramakrishnan et al. (2016); Jylhävä et al. (2017); Dugué et al. (2018); Peters et al. (2018); Zheng et al. (2018)
	PCa	Wang et al. (2007); Gurel et al. (2008); Hagelgans et al. (2013); Tang et al. (2013); Dumache et al. (2014); Litovkin et al. (2015); Gurioli et al. (2016); Jylhävä et al. (2017); Kim et al. (2017); Dugué et al. (2018); Giannopoulou et al. (2019)
Proteostasis	BCa	Rappa et al. (2012); Li et al. (2013); White (2015)
	RCa	Walker and Lithgow (2003); Gęgotek and Skrzydlewska (2019)
	PCa	Santarosa et al. (1997); Delie et al. (2013); Li et al. (2013); Li et al. (2019); Chen and Cubillos-Ruiz (2021)
Mitochondria dysfunction	BCa	Roumeguère et al. (2017); Mollo et al. (2020); Shorning et al. (2020)
	RCa	Echtay et al. (2003); Singh and Kulawiec (2009); Gęgotek and Skrzydlewska (2019); Mollo et al. (2020)
	PCa	Zarkovic (2003); Singh and Kulawiec (2009); Pal and Quinn (2013); Parr et al. (2013); Oh et al. (2016); Reznik et al. (2016); Mahalingaiah et al. (2017)
Cellular senescence	BCa	Chen et al. (2005); Toso et al. (2014); Gorgoulis et al. (2019)
	RCa	
	PCa	

BCa: bladder cancer; RCa: renal cancer; PCa: prostate cancer.

In addition to the direct effect of ALDH2 polymorphism, ALDH2-related aldehyde metabolites are also closely associated with cancers. 4-HNE was found to exhibit both endogenous carcinogenesis and antitumor effects (Zhang and Fu, 2021). Elevated oxidative stress and increased ROS generation have been confirmed to be related to a majority of cancer types by a large amount of strong evidence (DeNicola et al., 2011; Bellot et al., 2013), and 4-HNE generated from oxidative stress-induced lipid peroxidation plays a major role in the carcinogenic effects of lipid peroxidation (Zhong and Yin, 2015). In addition, p53 mutation induced by 4-HNE-DNA adducts is also one of the carcinogenetic mechanisms (Hu et al., 2002). Remarkably, the association between 4-HNE and early-stage carcinogenesis was first described in the case of renal cancer (RCa) (Segura-Aguilar et al., 1990). Interestingly, the antitumor effects of 4-HNE are linked to its concentration and cell type (Ayala et al., 2014). At a physiological or lower concentration, especially when it is similar to those in human tissues (Esterbauer et al., 1991), 4-HNE stimulates gene expression (especially Nrf2) to strengthen the antioxidant capacity of cells, prevent inflammation, and promote the adaptive response of immune cells for maintenance of homeostasis (Marantos et al., 2008). However, with concentration increasing, 4-HNE will tend to promote organelle and protein damage and thus inhibit cell proliferation and angiogenesis (Stagos et al., 2009). Simultaneously, it will induce apoptosis, differentiation, autophagy, and cellular senescence (Barrera et al., 2008; Pizzimenti et al., 2010). At a much higher level, 4-HNE will promote apoptosis or necrosis programmed cell death to avoid cancerization and eventually lead to cell death (Ayala et al., 2014). In this process, 4-HNE elicited its antitumor effect by regulating oncogenic signaling pathways and the expression of key genes, such as oncogenes c-myc (Fazio et al., 1992; Rinaldi et al., 2000; Pizzimenti et al., 2006), c-myb (Barrera et al., 1996), cyclin D, cyclin A (Skorokhod et al., 2010), and Notch1 (Pizzimenti et al., 2008). Moreover, the Doorn team confirmed that 4-HNE was not only a substrate but also an inhibitor of ALDH2, the inhibitory effect of which was reversible at a low concentration until 10 µM (Doorn et al., 2006). Therefore, ALDH2 inactivation induced by 4-HNE may play an essential role in the progression of some cancer species. As mentioned above, the effects of 4-HNE also depend on the cell type. For example, in hepatic cells, 4-HNE was found to promote p53 mutation and result in tumorigenesis, while it induced p53 expression in neuroblastoma cells, thus regulating cell cycle arrest or apoptosis induction and ultimately playing an antitumor role (Hu et al., 2002; Laurora et al., 2005). A similar phenomenon occurred in the regulation of NF-κB signaling pathway by 4-HNE (Ayala et al., 2014). Meanwhile, Lee et al. reported that long-term therapy with 0.1 µM 4-HNE led to an increase in cell growth in young smooth muscle cells (SMCs) but showed cytotoxicity to aged SMCs (Lee et al., 2006). In particular, 4-HNE is now believed to help normal cells defend cancer invasion and is more toxic to a variety of both hematological and solid tumor cells than normal cells. The differential effects of 4-HNE may be the consequence of changes in aldehyde-metabolizing enzymes, antioxidant defenses, and mitochondrial function (Pizzimenti et al., 2010; Barrera, 2012; Gasparovic et al., 2017).

**FIGURE 1:**
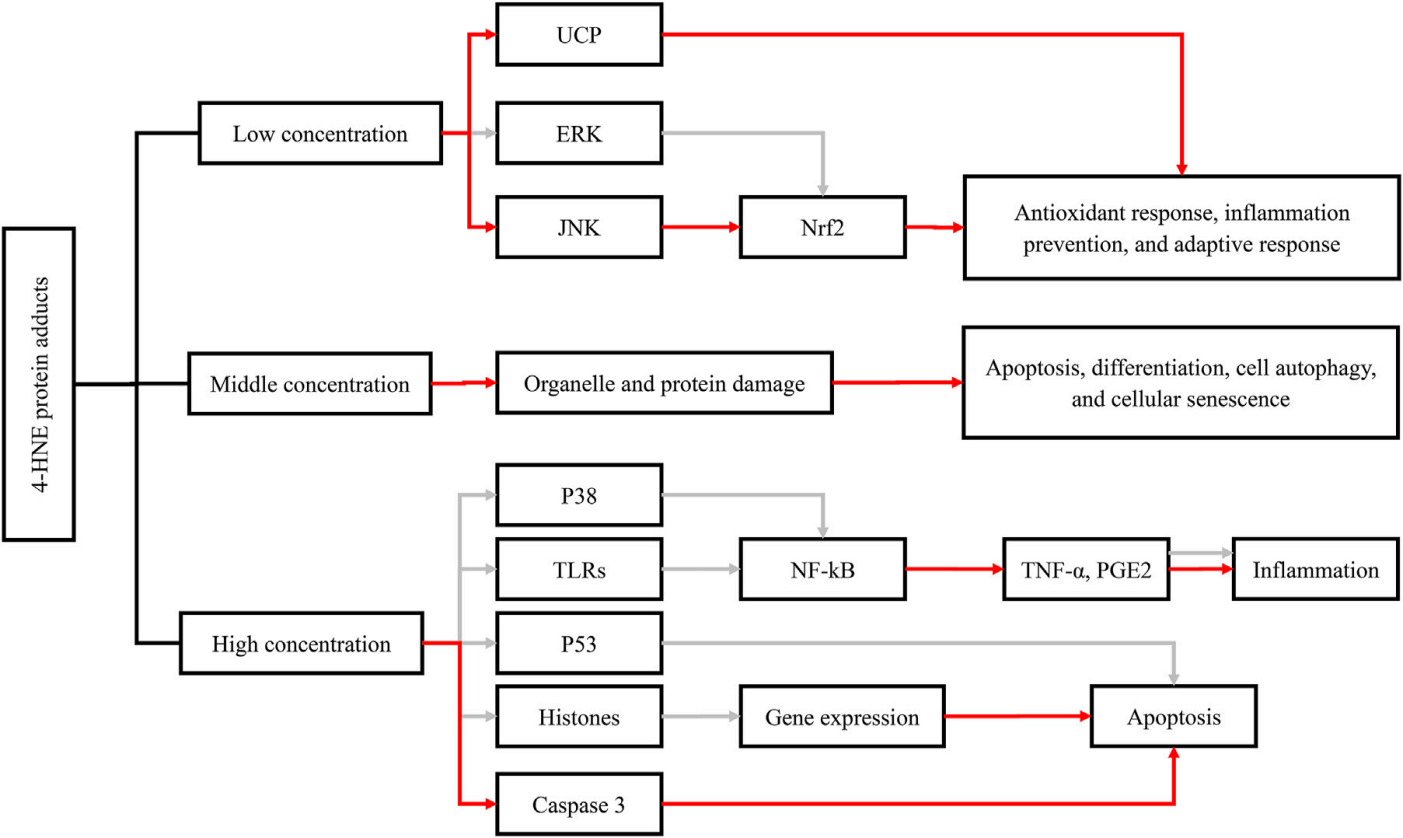
Functions of 4-HNE-protein adduct formation and their effects on cellular metabolic pathways, including the antioxidant response, inflammation, and apoptosis. The red arrow represents activation; the gray arrow represents inhibition. Abbreviations: 4-HNE, 4-hydroxynonenal; UCP, uncoupling protein; ERK, extracellular signal–regulated kinase; JNK, c-Jun N-terminal kinase; NFκB, nuclear factor kappa B; Nrf2, nuclear factor (erythroid-derived 2)-like 2; p38, protein 38; p53, protein 53; TLRs, toll-like receptors; TNF-α, tumor necrosis factor-α; PGE2, prostaglandin E.

Moreover, other reactive aldehydes, such as MDA and acrolein, also have similar bioactivities to 4-HNE. MDA was found to contribute to DNA damage and mutation (Niedernhofer et al., 2003; VanderVeen et al., 2003), and MDA-DNA adducts may ultimately lead to cell cycle arrest (Ji et al., 1998) and apoptosis (Willis et al., 2004) when DNA repair mechanism is lacking. Furthermore, acrolein seemed to play similar roles in carcinogenesis as HNE. Strikingly, acrolein was found to be associated with PCa progression and biochemical recurrence after prostatectomy and could be regarded as an excellently predictive biomarker of PCa relapse with an accuracy of approximately 90% (Custovic et al., 2010) (Figure 1).

**FIGURE 2:**
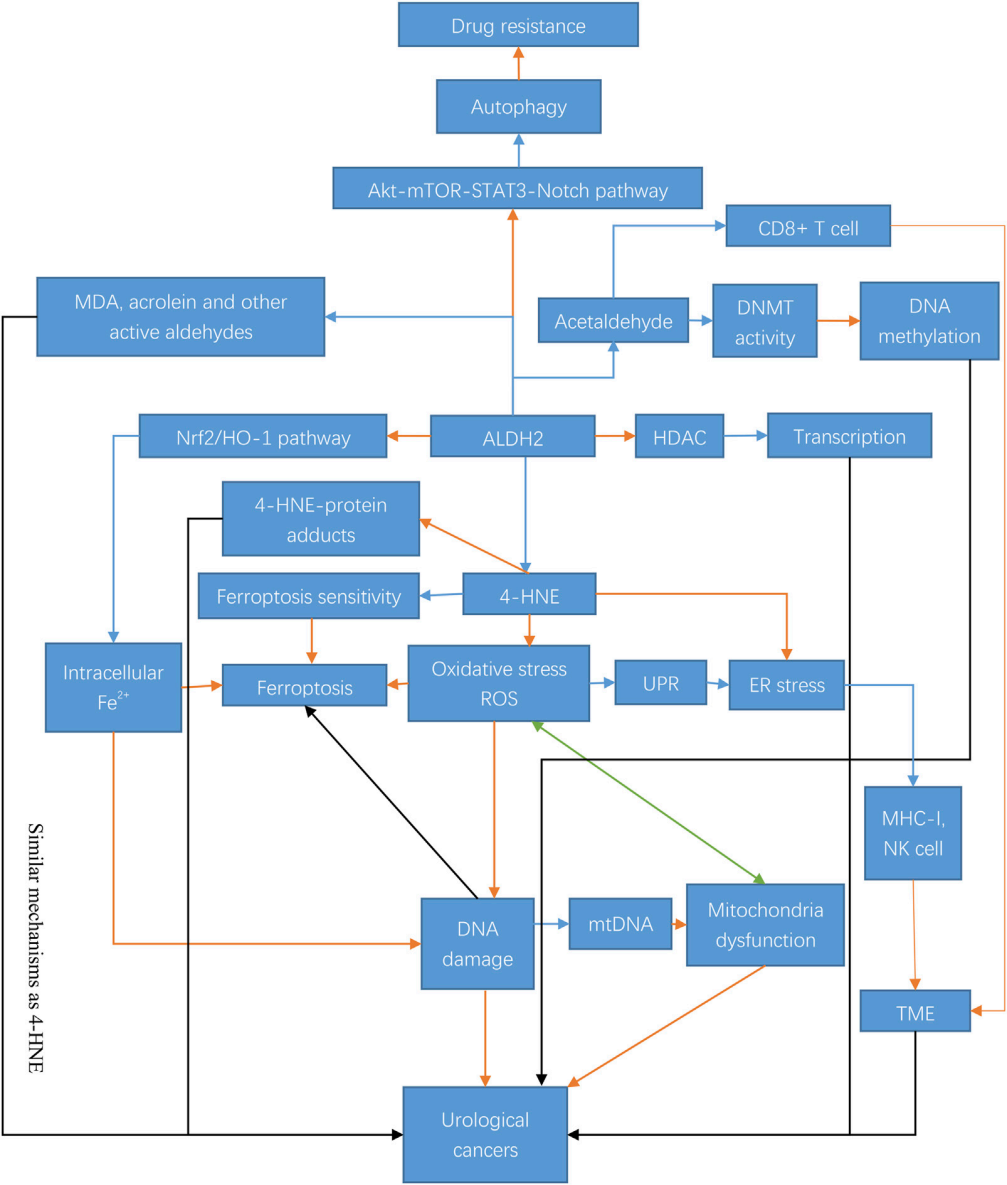
Mechanisms and regulation of ALDH2 in urological cancer tumorigenesis. ALDH2 exerts its antitumor effects in urological tumors through a variety of mechanisms. ALDH2 can play a regulatory role through epigenetic modifications and some pathways, such as the Akt-Notch pathway, and it can also regulate the generation of its metabolites, including 4-HNE, MDA, and acrolein, and play an antitumor role through ferroptosis, oxidative stress, autophagy and many other pathways. The blue arrow indicates inhibition; the orange arrow indicates activation; the black arrow indicates both activation and inhibition; and the green arrow indicates mutual interaction. Abbreviations: STAT3, signal transducer and activator of transcription-3; mTOR, mammalian target of rapamycin; DNMT, DNA methyltransferase; HO-1, heme oxygenase; HDAC, histone deacetylase; ROS, reactive oxygen species; UPR, unfolded protein response; ER stress, endoplasmic reticulum stress; MHC-I, major histocompatibility complex-I; mtDNA, mitochondrial DNA; TME, tumor microenvironment.

In summary, both ALDH2 and its associated metabolites are more or less directly or potentially related to cancer (Figure 2).

### Ferroptosis

As aging progresses, a disintegrated genome will lead to intracellular iron retention, resulting in DNA and epigenome damage, and contribute to genomic destabilization, which is one of the hallmarks of both aging and cancer. At the same time, DNA repair process induced by p53 is also blocked, and incomplete DNA further accelerates aging. This vicious cycle is called ferrosenescence (Sfera et al., 2018). In the process of ferrosenescence, retained iron leads to lipid peroxidation and endoplasmic reticulum (ER) stress, accompanied by cellular antioxidation failure, and ultimately results in ferroptosis (Sfera et al., 2018). However, a recent study showed that senescent cells with excessive accumulation of iron did not initiate ferroptosis but protected themselves from ferroptosis by avoiding the body’s self-renewal mechanism and accelerating body aging (Masaldan et al., 2018). Iron has been proven to be associated with RCa, PCa and BCa (Torti and Torti, 2013), in which the intracellular free iron concentration increased abnormally. An RCa model also described the carcinogenesis of iron overload by promoting DNA damage (Ebina et al., 1986). In aging and cancer, ALDH2 may be a double-edged sword. On the one hand, ALDH2 favor the antineoplastic effect of ferroptosis by lowering the 4-HNE concentration, and was also found to activate the Nrf2/HO-1 cascade to decrease intracellular iron (Ma et al., 2018). These effects may reveal the protective effect of ALDH2 at the initial stage of aging and carcinogenesis. On the other hand, it was reported that urological cancer was sensitive to ferroptosis inducers (FIN) (Yu et al., 2017), which indicates that ALDH2 may be a curative target for treatment-resistant PCa (Ghoochani et al., 2021). Therefore, reasonable activation of ferroptosis may be beneficial for both antiaging and antitumor effects. (Tao et al., 2019) reported that ALDH2*2 (rs671) male carriers had lower serum ferritin levels and that rs671 was significantly associated with ferritin concentrations. Accordingly, ALDH2 may regulate iron metabolism and thus contribute to ferroptosis. Different genotypes of ALDH2 among different populations may correspond to tumorigenic effect of alcohol in different degree, which could partly explain the contradictions on whether alcohol is a risk factor for urinary cancers. Furthermore, as a metabolite of lipid peroxidation, 4-HNE also has a strong connection with ferroptosis. Wang et al. reported that compared to low-stage cancers and normal tissues, the level of 4-HNE was relatively lower in high-stage cancer, and lower 4-HNE level was also associated with higher ferroptosis sensitivity in tumors (Wang et al., 2021). Previous studies also confirmed that tumors with a higher degree of malignancy were more sensitive to ferroptosis, manifesting higher propensity to metastasis and drug resistance, which indicated a potential antitumor role of 4-HNE (Viswanathan et al., 2017; Wu et al., 2019; Elgendy et al., 2020).

Given that ferroptosis acts specifically on malignant cells and protects normal cells, ferroptosis-targeted treatment has great therapeutic prospects for clinical applications. Precise therapy targeting ALDH2 and 4-HNE could be potential treatment approach for promoting ferroptosis sensitivity and inducing cell death and eventually achieve better prognosis among urological cancer patients.

## Epigenetic Alterations

### DNA Methylation

DNA methylation, as a common epigenetic mechanism of gene regulation, has been widely studied for the past few years. It is mainly mediated by DNA methyltransferases (DNMTs). Hypermethylation and hypomethylation in the promoter region is associated with gene silencing and transcription, respectively. Aging is the leading reason for the increased frequency of hypermethylation in CpG islands and promoters in many genes. Increasing global hypomethylation is associated with an increase in age, while many tumor suppressor genes are still hypermethylated (Maegawa et al., 2010; López-Otín et al., 2013). In addition, it is well established that local inflammation can predispose normal tissues to cancer, in which DNA methylation may have been involved (Easwaran et al., 2014). Modeled according to DNA methylation level, DNA methylation (DNAm) age, i.e., epigenetic age, is a more accurate predictor of human aging and biological age than telomere length (Jylhävä et al., 2017). DNAm age is strongly related to the occurrence and development of cancer. A meta-analysis found that accelerated DNAm age might increase the risk of death and adversely affect survival outcomes of RCa, PCa, and urothelial cancer (Dugué et al., 2018). Another study also found that DNAm age acceleration was highly correlated with genetic mutations in RCa and PCa (Horvath, 2013). For RCa, hypermethylation in genes such as TGF-β/RUNX3, NELL1, and ECRG4 can promote cell proliferation, EMT, tumor progression, migration, invasion, and metastasis of RCa (Ito and Miyazono, 2003; Mabuchi et al., 2010; Luo et al., 2016; Kim et al., 2017; Peters et al., 2018; Zheng et al., 2018). In PCa, hypermethylation of RARβ, cyclin D2 (CCND2), GSTP1, MGMT (Tang et al., 2013; Dumache et al., 2014; Litovkin et al., 2015; Gurioli et al., 2016) and hypomethylation of MYC, uPA, PLAU, S100P (Wang et al., 2007; Gurel et al., 2008; Hagelgans et al., 2013), may promote cancer cell proliferation, progression and metastasis and lead to poor clinical outcomes. (Jerónimo et al., 2004) showed that retinoic acid receptor beta2 (RARβ2) was hypermethylated in more than 90% of PCa and prostate intraepithelial neoplasia (PIN) cases while in only 20% of benign prostatic hyperplasia patients. Therefore, hypermethylation of RA receptor genes may affect PC progression by mediating gene expression. Additionally, it was also reported that DNA methylation is highly expressed in patients with BCa and might be relevant for bladder carcinogenesis (Jordahl et al., 2020).

DNA methylation plays a significant role in both aging and urological cancer, and ALDH2 may be involved in aging and urological cancer by regulating DNA methylation directly or indirectly. Firstly, ALDH2 may directly regulate the expression of methylated genes at the transcriptional level to exert its antitumor effect. For example, the ALDH2 promoter was found to contain a retinoid response element, which might contribute to gene regulation (Pinaire et al., 2003), which may be one of the antitumor mechanism for ALDH2 in PCa. Furthermore, ALDH2 also regulates gene methylation levels through its metabolites. Acetaldehyde was found to inhibit DNMT activity (Garro et al., 1991), and compared to healthy controls, long-term drinkers had significantly reduced mRNA levels of DNMT3a and DNMT3b (Bönsch et al., 2006). Metabolites of ALDH2, such as acetaldehyde and 4-HNE, have been proven to influence the clinical characteristics of liver cancer, colorectal cancer, breast cancer and upper aerodigestive tract cancer by regulating DNA methylation levels (Varela-Rey et al., 2013). Therefore, we speculated that a similar mechanism existed in urological cancer. Metabolites of ALDH2 may also regulate downstream signaling pathways of methylated genes since a low concentration of 4-HNE was found to stimulate cell proliferation and cell migration by promoting the nuclear factor kappa B (NF-κB) signaling pathway, and the expression of cyclin D1 and c-Myc (Xu et al., 2017) and 4-HNE also induced the production of TGF-β (Yang et al., 2019), which meant ALDH2 activation might decrease cell proliferation, migration, invasion, and EMT by reducing the concentration of 4-HNE.

### Histone Modification

Changes in histone modifications are important in the aging process (Muñoz-Najar and Sedivy, 2011). The acetylation levels of histones are affected by the catalytic equilibrium of histone acetyltransferases (HATs) and histone deacetylases (HDACs). In HDACs, the activity of the sirtuin family is directly linked to biological life cycle control. In aging cells, decreased SIRT1 activity leads to increased genomic aneuploidy and instability (Fatoba and Okorokov, 2011). In addition, SIRT6 depletion is associated with telomere dysfunction, resulting in chromosomal fusions and premature cellular senescence (Michishita et al., 2008). Further study showed that SIRT6 could participate in the antiaging process by reversing the effect of the NF-κB pathway (Kawahara et al., 2009). Additionally, as a component of DNA-dependent protein kinase, SIRT6 can directly participate in DNA damage repair by exerting homologous recombination and nonhomologous terminal junctions, maintaining genome stability and inhibiting the aging process (Mao et al., 2011). In RCa, it was reported that the overexpression of HDAC1 and HDAC6 both increased cell invasion and motility through increasing the expression of matrix metalloproteinase (Ramakrishnan et al., 2016). It was also reported that combining an HDAC inhibitor with an HIV protease inhibitor was effective for RCa cells (Sato et al., 2012). For BCa, HDAC1 mRNA was significantly overexpressed in BCa compared with normal tissues according to a small-scale study (Pinkerneil et al., 2017). While another study confirmed that HDAC1 and HDAC2 double knockout impaired cell proliferation function and led to apoptosis of urothelial carcinoma cells, which indicated the vital contribution of HDAC to BCa tumorigenesis and development (Pinkerneil et al., 2016). HDAC inhibition could induce a strong response of drug-resistant BCa cells (Juengel et al., 2017) and could exert adhesion-blocking properties on BCa cells (Juengel et al., 2013), which might also reveal a tumor-promoting effect of HDAC in BCa. Many studies have also described the oncogenic role of SIRT1-3 and the tumor suppressor role of SIRT4 and 6 in BCa (Giannopoulou et al., 2019). In PCa, it was observed that increased activity of HDACs was associated with elevated levels of serum PSA and increased invasiveness of tumor cells (Damaschke et al., 2013).

ALDH2 also participates in the process of aging and urological cancers by regulating histone modification. Several studies confirmed that ALDH2 could modulate SIRT1-mediated senescence by reducing the amount of 4-HNE (Gu et al., 2013; Xue et al., 2018). Furthermore, Choi et al. reported that ALDH2 could play an epigenetic regulatory role in gene translocation. It could bind to HDACs and result in higher HDAC activity, suggesting that ALDH2 would induce transcriptional repression (Choi et al., 2011). To sum up the above, we come to the conclusion that ALDH2 can inhibit the proliferation and development of urological cancer cells.

As a hallmark of aging, epigenetic alteration occurs not only in tumor cells but also in immune and stromal cells located in the tumor microenvironment (TME) by immune editing and reprogramming (Liu et al., 2017). Tumor cells escape elimination from the immune response through numerous mechanisms, including inhibiting the expression of genes related to tumor-associated antigens and antigen processing by DNA methylation and histone deacetylation (Dunn et al., 2017). In our previous study, we found that the number of CD8^+^ T cells, B cells, neutrophils and macrophages was positively correlated with the expression of ALDH2 in PCa and that ALDH2 could regulate the immune TME and decrease the inhibition of CD8^+^ T-cell activation and proliferation by reducing acetaldehyde accumulation. Here, we speculated that ALDH2 could also participate in immune TME regulation by not only its metabolites but also epigenetic alterations including DNA methylation and histone acetylation, while the latter is associated with human aging.

### Proteostasis

It is well accepted that aging and some aging-related diseases including varies urological cancers are associated with the loss of proteostasis (Powers et al., 2009). The heat shock response (HSR), unfolded protein response (UPR) and other mechanisms all help maintain protein homeostasis by assisting protein folding correctly and eliminating proteins that fail to fold. Lab animals overexpressing molecular chaperones was reported to possess an extensive life span (Walker and Lithgow, 2003; Morrow et al., 2004). Meanwhile, autophagy and other processes play an equally important role in protein homeostasis by cleaning and recycling garbage proteins through protein degradation. Significantly upregulated heat shock protein levels were observed in RCa (Santarosa et al., 1997), PCa (Rappa et al., 2012), and BCa (Ischia and So, 2013). However, the exact effect of ALDH2 on HSR in both aging and urological cancers is still unknown. It was reported that lipid peroxidation products could bind to HSPs to inhibit their degeneration; therefore, ALDH2 might act as an aging-promoting factor. However, ALDH2 may play a role in the antitumor process by inhibiting the function-enhancing extracellular release of HSP70 of 4-HNE (Yang et al., 2019). 4-HNE can bind to a variety of peptides and proteins, including glutathione, carnosine, enzymes, proteins on membranes and cytoskeleton, chaperones, uncoupling proteins (UCPs) in mitochondria, and antioxidant proteins to form 4-HNE-protein adducts (Poli et al., 2008; Ischia and So, 2013; Zhao et al., 2014). Approximately one-third of the binding target proteins are located in mitochondria (Poli et al., 2008; Zhao et al., 2014). 4-HNE-protein adducts affect the functions and bioactivities of these proteins. After that, adducts of 4-HNE and cyclin-dependent kinases alter enzyme activity, contributing to cell cycle delay (Camarillo et al., 2016). Adducts of 4-HNE with extracellular signal–regulated kinases also changed the function of 4-HNE, resulting in a decrease in Nrf2 activity and a loss of signal transduction, resulting in disorders in cellular homeostasis and cell proliferation (Lin et al., 2014). 4-HNE also adheres to histones and elongation factor-2, modifying gene expression at the transcriptional and translational levels, respectively (Gęgotek and Skrzydlewska, 2019). Furthermore, when bound to other proteins, such as Toll-like receptors, 4-HNE could mediate immune regulation (Gęgotek and Skrzydlewska, 2019). Previous studies have confirmed that 4-HNE protein adducts formed in RCa tissues are related to cancer proliferation and progression (Shoeb et al., 2014).

The TME, partly characterized by high metabolism, hypoxia, nutrition limitation, and acidosis, changes the protein processing capacity of the ER of both cancer cells and infiltrating immune cells, thus leading to accumulation of errant proteins and eventually ER stress. A persistent, yet moderate ER stress response promotes cancer cell proliferation, invasion, metastasis, drug resistance, angiogenesis and immune escape through several mechanisms (Chen and Cubillos-Ruiz, 2021), and cells respond to ER stress by activating the UPR. As a marker of ER stress, GRP78 is overexpressed in PCa and BCa, which is related to tumor development, recurrence and a poor survival outcome (LeRoy et al., 2007; Delie et al., 2013; Li et al., 2013). ALDH2 may inhibit urological cancers by regulating ER stress. For instance, it was found to strengthen the UPR by reducing oxidative stress (Long et al., 2020), and increased expression of ALDH2 was associated with reduced ER stress (Yan et al., 2020). The ER stress of cancer cells can also influence tumor progression by changing the function of the immune TME. It was reported that induction of ER stress impaired major histocompatibility complex class I-peptide (MHC I-peptide) presentation (Granados et al., 2009), and it altered NK-cell-mediated recognition of tumors (Obiedat et al., 2019). Therefore, ALDH2 may also possess an antitumor effect by regulating the TME.

Currently, as a nexus of aging and cancers, autophagy can determine key physiological decisions from cell fate to body lifespan through metabolism and proteostasis pathways. Autophagy works by degrading defective mitochondria and thus provides extra energy, including fatty acids and glutamine, for tumor cells (White, 2015). The blockade of autophagy significantly decreased drug-induced resistance in BCa (Li et al., 2019) and PCa (Farrow et al., 2014). The mTOR and AMP kinase (AMPK) signaling pathways are also significantly involved in aging and cancer. Upregulated mTOR signaling was confirmed to be associated with the development, progression and metastasis of PCa (López-Otín et al., 2013), and downregulated mTOR extended the lifespan of many kinds of laboratory animals (Zaytseva et al., 2012). It was reported that overexpression of ALDH2 suppresses autophagy (Wang and Wu, 2019), possibly by restoring the Akt-mTOR-STAT3-Notch signaling cascade (Ge and Ren, 2012), and ALDH2 might play a repressive role in transcriptional control by AMPK activation (Choi et al., 2011). Thus, ALDH2 could be a possible therapeutic target for both aging and urological cancer.

### Mitochondrial Dysfunction

A decline in mitochondrial function occurs with aging. Many studies have shown that mtDNA increases with age in the human body (Corral-Debrinski et al., 1992; Fayet et al., 2002). Experimental evidence revealed the exact relationship between mtDNA and aging, which showed that the accumulation of mtDNA mutations could result in a premature aging phenotype (Trifunovic et al., 2004). In addition to primary mitochondrial dysfunction, abnormal mitochondrial biogenesis, secondary to abnormalities in nuclear genes and mitochondrial metabolism-dependent factors, such as ROS, NO, NAD+/NADH, ATP, and Ca2^+^, also promotes aging (Ryan and Hoogenraad, 2007). SIRT3 can help maintain mitochondrial and cellular homeostasis by regulating the ROS impacts of numerous physiologies linked with aging (van de Ven et al., 2017). The PI3K/Akt/mTOR signaling pathway is also involved in the regulation of mitochondrial biosynthesis, autophagy and apoptosis. The increase in mTOR activity in aging cells not only results in the accumulation of damaged mitochondria by inhibiting mitochondrial autophagy but also leads to mismatched production of mitochondria and metabolites by improving cell metabolic activity, thus aggravating the damage to mitochondrial function (Mollo et al., 2020).

Compared to normal tissues, there was a tendency of depletion in mtDNA in RCa and BCa (Reznik et al., 2016), especially in PCa (Parr et al., 2013). For example, the mtDNA G10398A polymorphism was related to a higher risk of PCa (Singh and Kulawiec, 2009). Many studies have also shown the significance of oxidative stress in the malignant transformation of kidney epithelial cells (Mahalingaiah et al., 2017), PCa tumorigenesis (Oh et al., 2016; Roumeguère et al., 2017), and the etiology and progression of BCa (Islam et al., 2019). It is widely known that since 1993,4-HNE has been regarded as a “toxic product of lipid peroxidation” and a “second toxic messenger of free radicals,” taking part in several signaling pathways related to proliferation, cell cycle arrest, apoptosis, and the regulation of the expression of a large number of genes (Cheng et al., 2001; Yang et al., 2001; Sharma et al., 2004; Vatsyayan et al., 2011), and has become a reliable biomarker of oxidative stress (Zarkovic, 2003). 4-HNE is also involved in the progression of RCa through oxidative stress-associated mechanisms (Shoeb et al., 2014). Therefore, modulation of 4-HNE-associated oxidative stress and 4-HNE-associated mitochondrial dysfunction can be utilized as a treatment method in cancer prevention and treatment. Existing studies have suggested that 4-HNE may decrease ROS and its related production and reduce oxidative damage in cancer by activating UCPs (Echtay et al., 2003; Esteves and Brand, 2005; Valle et al., 2010). Moreover, strong 4-HNE formation can affect mitochondrial function and ultimately lead to cell death (Zhao et al., 2014).

Overactivation of PI3K-Akt-mTOR signaling also promoted RCa cell proliferation, migration and metastasis, as well as angiogenesis and treatment resistance (Pal and Quinn, 2013) through the same mechanism as PCa (Shorning et al., 2020) and BCa (Xu et al., 2020). A decrease in mitochondrial detoxification induced by lipid peroxidative aldehydes has been observed and was hypothesized as a potential mechanism of aging in animal models (Yu, 2005; Ohsawa et al., 2008). Since ALDH2 is a mitochondrial chaperone (Chen et al., 2014; Wang et al., 2020) confirmed that ALDH2 deficiency might be linked to mtDNA accumulation in the cytoplasm, which indicated that mtDNA damage and ALDH2 recruitment preserve mitochondrial integrity. Elevated oxidative stress products (such as MDA), secondary to ALDH2 loss, not only mediate mitochondrial dysfunction, leading to mtDNA damage and contributing to cellular senescence and aging-associated phenotypes (Gosselin et al., 2009) but also promote cancer development through damaged mtDNA in hepatocellular carcinoma (Seo et al., 2019). It was shown in a preclinical model that the ability of ALDH2 to degrade 4-HNE declines with aging (Chen and Yu, 1996). Therefore, ALDH2 activation may slow down the aging process. Although the role of this mechanism of ALDH2 in the occurrence and development of urological cancers is still unknown, we assumed a similarity here. The interaction between SIRT3 and ALDH2 affects ALDH2-NAD+ binding (Harris et al., 2017), which indicates that ALDH2 might inhibit urological cancer by regulating ROS and oxidative stress.

### Cellular Senescence

Cellular senescence was first described by (Hayflick, 1965) as cell proliferative activity decreased with an increase in division number and ultimately cell cycle arrest and cell death, which was called replicative senescence. Recently, it has been pointed out that cell senescence is a kind of cell state generated by pressure signal stimulation and exists in specific physiological processes with four typical characteristics: cell cycle arrest, senescence-associated secretion phenotype (SASP), macromolecular damage and metabolic disorder (Gorgoulis et al., 2019). Cellular senescence is the basis of biological aging, and most single-cell senescence also has the same characteristics as aging. In addition to epigenetic alterations, proteostasis, and mitochondrial dysfunction, cellular senescence is involved in altered intracellular communication through SASP. Senescent cells play a dual role in promoting and attenuating cancer. On the one hand, senescent tumor cells were found in prostate intraepithelial neoplasia but not in the corresponding malignant stage (Chen et al., 2005). This evidence suggests that senescence is an obstacle to the malignant development of tumors and can effectively inhibit the malignant transformation of tumors. Activation of specific oncogenes or inactivation of tumor suppressor genes can induce the senescence process to help cell cycle arrest. For instance, senescent hallmarks were also detected in early-stage prostate tumors (Courtois-Cox et al., 2006). SASP also has antitumor effects. Some SASPs, such as IL-6 and IL-8, promote senescence through a positive feedback loop of autocrine signaling and reduce cell transformation and metastasis by inhibiting the activation of some oncogenes (Wajapeyee et al., 2008). However, on the other hand, SASP is more likely to play a role in carcinogenesis in PCa. It is mainly reflected in the following two aspects: 1) SASPs provide an immunosuppressive microenvironment for tumors and promote tumor immune escape. 2) SASPs promote tumor invasion and metastasis. Senescent cells suppress the antitumor immune response by inducing granulocytic myeloid-derived suppressor cell infiltration and thus inhibiting T cell activity (Toso et al., 2014). Levels of both IL-6 and sIL-6R were found to be strongly associated with bone metastases, tumor volume, and risk of progression in prostatectomy patients (Nguyen et al., 2014). IL-8 was found to induce FGF2 expression, promoting abnormal proliferation in the transition zone (Giri and Ittmann, 2001). Other SASPs, such as TGF-β1, CXCL12, MMP2, and MAPK, are also involved in this process (Fiard et al., 2021). Furthermore, SASPs have also been shown to decrease EMT in cancer cells (Coppé et al., 2008), and a phenotype has been shown to be resistant to chemotherapy and radiation in BCa (McConkey et al., 2009).

ALDH2 may inhibit stress-induced senescence by reducing some aldehyde metabolites of oxidative reactions. It was found that ALDH2 impairment accelerated the acquisition of a premature senescent phenotype mainly due to the impairment of mitochondrial bioenergetic functions and cellular communication (Nannelli et al., 2018). This evidence indicates a potential association between ALDH2 and senescence. NF-κB was activated and enriched in the chromatin portion of senescent cells (Acosta et al., 2008; Kuilman et al., 2008; Chien et al., 2011), regulating senescence by directly regulating SASPs, including IL-8 and IL-6, which in turn controlled SASP transcription and expression. mTOR also regulates SASPs by mediating the translation of MAP kinase–activated protein kinase 2 (MAPKAPK2) (Herranz et al., 2015). Therefore, for urological cancer, as mentioned before, ALDH2 may play an antitumor role in regulating SASPs such as TGF-β, IL-6, and IL-8 by regulating the NF-κB and mTOR pathways.

## Conclusion

ALDH2 might be involved in the development and progression of urological cancers through multiple cellular processes.
